# Microsatellite based genetic diversity and population structure of the endangered Spanish Guadarrama goat breed

**DOI:** 10.1186/1471-2156-10-61

**Published:** 2009-09-29

**Authors:** Magdalena Serrano, Jorge H Calvo, Marta Martínez, Ane Marcos-Carcavilla, Javier Cuevas, Carmen González, Juan J Jurado, Paloma Díez de Tejada

**Affiliations:** 1 Departamento de Mejora Genética Animal, INIA. 28080 Madrid, Spain; 2 Unidad de Tecnología en Producción Animal. CITA-Gobierno de Aragón. 727 50080 Zaragoza, Spain; 3 CENSYRA. IMIDRA, Consejería de Economía y Consumo, Comunidad de Madrid, Ctra. de Guadalix de la Sierra, Km, 2. 28770 Colmenar Viejo, Madrid, Spain; 4 Asociación de Criadores de Ganado Caprino de la Raza de Guadarrama, Ctra. de Guadalix de la Sierra, Km. 2. 28770 Colmenar Viejo, Madrid, Spain

## Abstract

**Background:**

Assessing genetic biodiversity and population structure of minor breeds through the information provided by neutral molecular markers, allows determination of their extinction risk and to design strategies for their management and conservation. Analysis of microsatellite loci is known to be highly informative in the reconstruction of the historical processes underlying the evolution and differentiation of animal populations. Guadarrama goat is a threatened Spanish breed which actual census (2008) consists of 3057 females and 203 males distributed in 22 populations more or less isolated. The aim of this work is to study the genetic status of this breed through the analysis of molecular data from 10 microsatellites typed in historic and actual live animals.

**Results:**

The mean expected heterozygosity across loci within populations ranged from 0.62 to 0.77. Genetic differentiation measures were moderate, with a mean F_ST _of 0.074, G_ST _of 0.081 and R_ST _of 0.085. Percentages of variation among and within populations were 7.5 and 92.5, respectively. Bayesian clustering analyses pointed out a population subdivision in 16 clusters, however, no correlation between geographical distances and genetic differences was found. Management factors such as the limited exchange of animals between farmers (estimated gene flow Nm = 3.08) mostly due to sanitary and social constraints could be the major causes affecting Guadarrama goat population subdivision.

**Conclusion:**

Genetic diversity measures revealed a good status of biodiversity in the Guadarrama goat breed. Since diseases are the first cause affecting the census in this breed, population subdivision would be an advantage for its conservation. However, to maintain private alleles present at low frequencies in such small populations minimizing the inbreeding rate, it would necessitate some mating designs of animals carrying such alleles among populations. The systematic use of molecular markers will facilitate the comprehensive management of these populations, which in combination with the actual breeding program to increase milk yield, will constitute a good strategy to preserve the breed.

## Background

Before the intensification and industrialisation process of the last decades, European livestock farming was generally extensive and closely linked to the use of farmland. This is still the case for small ruminants and especially for goats, where not only local breeds do not benefit from modern breeding techniques but they also are about to disappear. Thus, the decline of local breeds and their production systems are raising concern about the importance of European agro-ecosystems and cultural landscapes maintenance. The goat is among the earliest species to be domesticated [1]. Goats are distributed over all types of eco-niches, including tropical areas, dry zones and mountain regions. With such a wide distribution and adaptability [2], the goat is expected to have high genetic diversity as a result of both, natural selection for fitness under varied environmental conditions and the artificial selection for milk, meat, fibre and other purposes.

However, in contrast to high productive foreign goat breeds, most local breeds are not subject to breeding programs to improve production traits, which would increase their genetic ability for productivity and consequently their profitability. Due to the extensive conditions of animal management, existing breeding strategies applied to local breeds are constrained by poor pedigree recording. In this way, the lack of pedigree records can lead to both a limited genetic progress for the selected trait and a suboptimal inbreeding control. The use of highly variable molecular genetic markers, such as microsatellites, is one of the most powerful means for studying genetic diversity and pedigree reconstruction because of their high degree of polymorphism, random distribution across the genome and neutrality with respect to selection [3-5].

Guadarrama goat, a rustic breed which has been exploited in mountain areas in the centre of Spain since XVIII century, constitutes a good illustrative example. Guadarrama goat is a threatened breed whose actual population consists of 3057 females and 203 males distributed in 22 herds, which correspond to an effective population size of 763 individuals considering only the unequal number of males and females [6]. Other effects not considered here, as unequal parental contributions, overlapping generations, genetic drift, selection etc, can also influence the magnitude of the effective size. Common diseases such as tuberculosis and paratuberculosis are the main causes of the high culling rates (near 25% per year) existing in this breed. Generally, herd's size ranged from 300 to 500 animals.

This breed is mainly used for milk production, although it is also exploited in a local meat industry. Since 1998 Guadarrama breeders association has established a breeding program to increase milk yield. There are a high degree of disconnectedness among the herds of this breed and scarce pedigree information due to the low spread of the artificial insemination. Therefore, genetic evaluations of animals for milk yield are only comparable at the intra-herd level. For this reason, a pilot project for pedigree reconstruction based on molecular markers information (10 microsatellite) has been established in 2003 as an alternative to pedigree recording.

The aim of this work is to evaluate the genetic status of the Guadarrama goat breed making use of the molecular information generated in the breeding program analyzing both, their genetic diversity and its population structure or subdivision using clustering methods.

## Methods

### Biological samples

**Figure 1:**
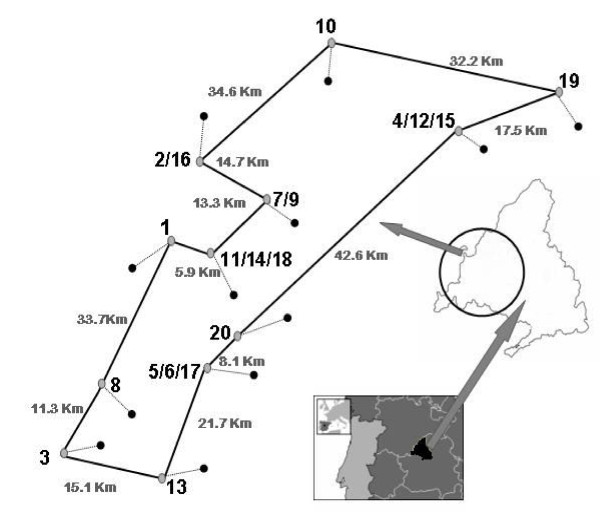
**Geographic distribution of the 20 Guadarrama goat populations analyzed**.

Fresh blood samples were obtained between years 2004-2007 from 6635 goats pertaining to 20 different herds, which constitutes the whole animals of the Guadarrama breed. Figure 1 illustrates geographical locations of these 20 populations. Total genomic DNA was isolated from blood using the Real Biotech Corporation ADN extraction kit (Durbiz).

### Genetic loci

Ten microsatellite markers were studied: ILSTS005 [7], CSSM31 [8], BM8125 [9], BM1818 [9], ILSTS011 [10], INRA006 [11], CSSM066 [12], RM006 [13], BM6526 [9] and MCM53 [14]. Some of them had been previously recommended in biodiversity studies by FAO and ISAG. Microsatellites amplification was carried out using fluorescent labelled primers. The amplified products were analysed with a DNA capillary sequencer ABI Prism^® ^310 Genetic Analyzer (Applied Biosystems).

### Statistical analysis

The expected heterozygosity corrected for sampling bias [15], the observed heterozygosity, the polymorphic information content and the estimated null allele frequency were calculated for each locus in the whole population using CERVUS version 3.0.3. [16]. GENEPOP 3.4 package [17] was used to perform the exact test for Hardy-Weinberg equilibrium by microsatellite loci (test multi-population) and by population (test multi-locus) using the Markov chain method with 1000 iterations, and considering the heterozygote deficit as the alternative hypothesis. Wright's *F-statistics *F_IS_, F_ST_, and F_IT _[18] jackknifing over populations and loci were calculated by FSTAT version 2.9.3.2 [19]. Gene flow (Nm) was estimated by the approximation of Wright [20] F_ST _≈ 1/(1+4Nm) assuming genetic markers neutrality and an island model. Heterozygosities, mean number of alleles across populations, F_IS _within populations and Gst were calculated with GENETIX 4.03 software [21]. Furthermore, F_ST _values for pairwise comparisons of the 20 Guadarrama goat herds and their significance level for genetic differentiation and Rst were tested with FSTAT. Significance levels were set using the sequential Bonferroni correction (initial k = 190). GENCLASS 2.0 package [22] under a Bayesian approach [23] was used in the assessment of animals to the predefined populations in which their respective genotypes were most likely to occur. The Mantel test [24] was performed with GENETIX 4.03 to test the correlation between the F_ST _values and the geographic distances between populations. The population structure was analyzed by cluster techniques with the software STRUCTURE 2.1 [25] and BAPS 4.14 [26,27]. Due to the high number of missing data for the BM1818 marker, only nine of the ten loci genotyped were used in these analyses. According to Falush et al. [28], STRUCTURE analysis was performed considering both the admixture model and the correlated allele frequencies between populations. The length of the burn-in and MCMC (Monte Carlo Markov chain) were 10,000 and 100,000, respectively. For the whole data set (6635 animals distributed in 20 original populations) 15 runs were carried out for each value of K, being K the number of clusters. The range of possible Ks tested was from 2 to 23 (the real number of herds plus 3). For each value of K the mean of the log probability of data (L(K)) over 15 runs were calculated. F_ST _mean values for each cluster were also estimated. BAPS was run setting the maximum number of cluster at 20. Results were based on 50 simulations from the posterior allele frequencies. Finally, locus by locus AMOVA analysis considering groups and populations as sources of variation was assessed by ARLEQUIN 3.1 software package [29].

## Results

### Microsatellite markers

A total of 170 alleles were detected at the 10 microsatellite loci assessed in the 6335 goats genotyped. Table 1 shows the genetic variability measures corresponding to these 10 loci. Differences in the number of animals genotyped per microsatellite were due to amplification failures. There were many problems with the amplification of the marker BM1818, which finally was genotyped only in 1371 animals. Except ILSTS005 (0.44), all markers were highly informative (PIC>0.50) which make them useful in genetic diversity studies. The number of alleles per locus ranged from 9 (ILSTS005) to 36 (CSSM66) being 17 the mean number of alleles per locus. Private alleles (UAN in table 1) occurred at very low frequencies (<0.025) for all loci in most populations. The mean observed and expected heterozygosities across loci were 0.70 (SD 0.09) and 0.77 (SD 0.10), respectively. Only the CSSM066 marker was characterized by a fairly high frequency of null alleles (11%).

**Table 1:** Genetic variability at the microsatellites typed in the Guadarrama goat breed.

**Locus**	**k**	**N**	**Ho**	**He**	**PIC**	**UAN**	**F(Null)**
ILSTS005	9	6484	0.491	0.504	0.442	3	0.0104
RM006	20	5819	0.762	0.798	0.774	3	0.0212
ILSTS011	11	6470	0.617	0.688	0.638	2	0.0563
BM1818	11	1371	0.640	0.766	0.739	2	0.0984
CSSM31	22	4750	0.782	0.865	0.851	2	0.0512
CSSM066	36	6439	0.667	0.843	0.828	6	0.1161
INRA006	17	4863	0.764	0.837	0.818	5	0.0452
BM6526	19	6498	0.771	0.818	0.798	2	0.0281
BM8125	9	6542	0.747	0.788	0.760	1	0.0258
MCM53	16	5635	0.785	0.810	0.785	2	0.0140

**k **= number of alleles at each locus; **N **= number of individuals typed for each locus; **Ho **= mean heterozygosity observed (direct count estimate); **He **= mean heterozygosity expected (unbiased estimate Nei, 1987); **PIC **= polymorphic information content; **UAN **= Unique allele number; **F(Null) **= Null allele frequency estimated.

Table 2 shows Wright' *F-statistics *and gene flow (Nm) for each locus across the 20 herds of Guadarrama goat breed. Mean values of F_IS _and F_ST _across loci were 0.023 and 0.074, respectively.

**Table 2:** Summary of Wright's *F-statistics *for each loci in the Guadarrama goat breed

**Locus**	**Sample size**	**F_IS _(S.E.)**	**F_IT _(S.E.)**	**F_ST _(S.E.)**	N_m_*
ILSTS005	12960	-0.023 (0.013)	0.031 (0.023)	0.052 (0.015)	4.558
RM006	11560	0.004 (0.009)	0.044 (0.009)	0.041 (0.006)	5.848
ILSTS011	12934	0.008 (0.009)	0.106 (0.031)	0.099 (0.028)	2.275
BM1818	2738	0.028 (0.015)	0.206 (0.085)	0.183 (0.083)	1.116
CSSM31	9438	0.034 (0.017)	0.104 (0.020)	0.073 (0.016)	3.175
CSSM066	12768	0.159 (0.015)	0.219 (0.014)	0.071 (0.005)	3.271
INRA006	9718	0.037 (0.014)	0.091 (0.017)	0.057 (0.014)	4.136
BM6526	12976	-0.010 (0.007)	0.063 (0.012)	0.072 (0.012)	3.222
BM8125	13082	-0.008 (0.010)	0.056 (0.017)	0.064 (0.014)	3.656
MCM53	11266	-0.030 (0.007)	0.034 (0.016)	0.063 (0.013)	3.718

All	10944	0.023 (0.018)	0.095 (0.020)	0.074 (0.011)	3.128

S.E. standard error

* Nm gene flow estimated from N_m _= 0.25(1- F_ST_)/F_ST _(Nei, 1987).

Wright's statistics according to Weir and Cockerham, 1984

Results of the Fisher's exact test for Hardy-Weinberg (HW) equilibrium across loci and populations, considering the heterozygote deficit as the alternative hypothesis, are shown in Table 3. Highly significant (p < 0.001) multilocus departures from HW proportions were found for most populations and significant (p < 0.05) for populations 2 and 20. Populations 10, 12 and 16, had non significant p-values for the statistical test. Single locus test across populations to asses departure from HW showed no significant p-values for ILSTS005, ILSTS011, BM6526, BM8125 and MCM53 markers.

**Table 3:** Hardy-Weinberg exact test in the Guadarrama goat populations.

**Population**	**Pop. Size**	**P-val**	**S.E.**	**Locus**	**P-val**	**S.E.**
1	720	0.000	0.000	ILSTS005	0.063°	0.006
2	147	0.049	0.009	RM006	0.000	0.000
3	411	0.000	0.000	ILSTS011	0.147°	0.012
4	517	0.000	0.000	BM1818	0.011	0.002
5	327	0.003	0.001	CSSM31	0.000	0.000
6	491	0.000	0.000	CSSM066	0.000	0.000
7	182	0.000	0.000	INRA006	0.000	0.000
8	218	0.004	0.002	BM6526	0.850°	0.020
9	272	0.000	0.000	BM8125	0.169°	0.017
10	141	0.805°	0.017	MCM53	0.998°	0.000
11	305	0.000	0.000			
12	362	0.192°	0.017			
13	687	0.000	0.000			
14	183	0.000	0.000			
15	570	0.000	0.000			
16	279	0.067°	0.010			
17	187	0.000	0.000			
18	154	0.000	0.000			
19	264	0.000	0.000			
20	217	0.040	0.007			

Markov chain parameters for all tests: Demorization: 1000; Batches: 100; Iterations per batch: 1000

° No significant departure from Hardy-Weinberg equilibrium.

Alternative hypothesis was heterozygote deficit

### Genetic diversity within populations

The mean number of alleles across loci, the mean observed and expected heterozygosities and the F_IS _estimates within the 20 Guadarrama goats populations, are shown in Table 4. Mean number of alleles across loci was higher than 9 in half of the populations. Heterozygosity deficit, as measured by Wright's F_IS_, was positive in most populations when averaged across loci, raging from -0.015 (population 10) to 0.066 (population 18). Average value of F_IS _across loci and populations was 0.022.

**Table 4:** Genetic diversity measures in each population of the Guadarrama goat breed.

**Population**	**Nall**	**H_obs_**	**H_exp_**	F_IS_**(IC 95%)
1	10.70	0.724 (0.091)	0.745 (0.090)	0.028 (0.012-0.043)
2	8.20	0.744 (0.163)	0.741 (0.140)	-0.005 (-0.103-0.018)
3	11.30	0.726 (0.120)	0.741 (0.130)	0.020 (-0.000-0.038)
4	7.60	0.665 (0.115)	0.697 (0.117)	0.045 (-0.007-0.091)
5	10.20	0.764 (0.093)	0.777 (0.103)	0.016 (-0.010-0.037)
6	10.20	0.714 (0.095)	0.746 (0.096)	0.042 (-0.000-0.071)
7	6.00	0.587 (0.118)	0.627 (0.141)	0.062 (-0.017-0.119)
8	9.30	0.719 (0.097)	0.749 (0.120)	0.041 (-0.001-0.075)
9	9.40	0.716 (0.119)	0.745 (0.127)	0.038 (0.011-0.062)
10	7.70	0.765 (0.114)	0.753 (0.082)	-0.015 (-0.056-0.014)
11	9.80	0.695 (0.125)	0.728 (0.117)	0.047 (-0.013-0.090)
12	7.80	0.663 (0.176)	0.659 (0.158)	-0.006 (-0.031-0.015)
13	13.00	0.754 (0.114)	0.765 (0.114)	0.014 (-0.003-0.029)
14	9.40	0.719 (0.085)	0.716 (0.099)	-0.004 (-0.046-0.023)
15	7.60	0.653 (0.133)	0.658 (0.125)	0.007 (-0.010-0.022)
16	8.70	0.647 (0.262)	0.655 (0.264)	0.011 (-0.013-0.032)
17	8.50	0.714 (0.092)	0.750 (0.080)	0.047 (0.005-0.080)
18	6.90	0.622 (0.166)	0.667 (0.114)	0.066 (-0.023-0.118)
19	11.60	0.724 (0.177)	0.760 (0.183)	0.048 (0.022-0.069)
20	6.80	0.688 (0.217)	0.700 (0.213)	0.017 (-0.045-0.050)

Nall = Mean number of alleles per loci; H_obs _= mean observed heterozygositiy; H_exp _= Nei's (1978) unbiased expected heterozygosity. Standard deviation in brackets

** 10000 Bootstrap over F_IS _by population, IC 95% = confidence interval at 95%

F_ST _values of pair-wise comparisons among the 20 herds (matrix not shown) of Guadarrama goats, showed an overall genetic differentiation F_ST _of 0.074 (SD 0.011) and pair-wise F_ST _values ranging from 0.027 (pop13 vs. pop19) to 0.165 (pop12 vs. pop20). Significant (α = 0.05) genetic differentiation was found after sequential Bonferroni correction (initial k = 190) in 92 out of 190 population pairs.

Results from GENECLASS assignment test revealed that about 77% of the animals were assigned to the population they were collected from. The higher percentages (91.2% to 97.2%) of individuals assigned to its original population occurred in populations 7, 10, 15, 18 and 20 while the lower correspond to populations 4 (68.2%) and 19 (69.0%). Assignment of individuals was consistent with the extent of genetic divergence reported in the F_ST _analysis. The marker MCM53 showed two private alleles, which were present only in population 15. The marker BM8125 had only one private allele found in population 1. On the other hand, populations 4, 12, 14 and 17 did not show private alleles at any microsatellite analyzed.

The Mantel test including the 20 populations of Guadarrama goats and the relative distances among them (Figure 1), depicted no significant correlation between the F_ST _values and the geographical distances (r = 0.105, p = 0.468).

### Population structure

**Figure 2:**
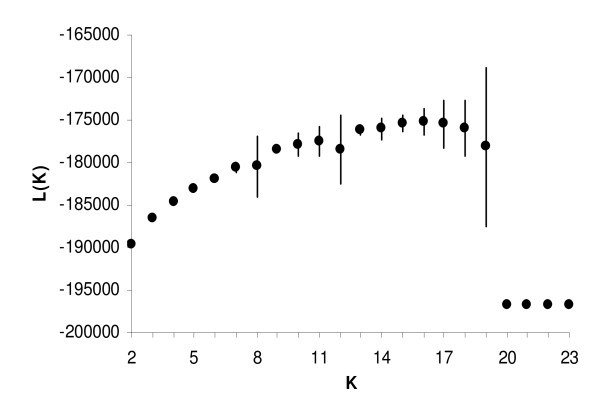
**Log probability of data (L(K)) for K values ranging from 2 to 23 for the admixture and correlated frequencies model, under exhaustive sampling (averaged over 15 replicates) for the Guadarrama goat breed (K = number of clusters)**. Length of burn-in 10,000. MCMC 100,000 Vertical bars reflect standard deviations.

Figure 2 shows the log probability of data (L(K)) for the admixture and correlated frequencies model under exhaustive sampling (averaged over the 15 replicates) of the STRUCTURE package. The highest L(K) averaged over replicates running for each value of K (K from 2 to 23), was observed for K = 16 (-175,201.08). For K varying from 2 to 17 and from 20 to 23 the runs reach equilibrium and converged to similar L(K) values. However for K equal to 18 and 19, the system showed more erratic values across replicates. Therefore for these values of K, 10 new replicates were made setting the length of the burning period in 50,000 and of the MCMC in 500,000. Similar but more stable values of L(K) were found in this case.

Estimated α values averaged 0.04 for K varying from 2 to 19, indicating that most individuals were essentially from one population or another. However, for K values ranging from 20 to 23, α values varied from 1.50 to 2.50 indicating that most individuals were admixed.

Using BAPS package the highest likelihood was also obtained with K = 16 (-174,885.14).

Table 5 shows the percentage of membership for each predefined population and the mean value of F_ST _in each of the 16 inferred clusters, for the high estimate of L(K) (-174,478.30) among the 15 replicates ran for K = 16. Clusters 2, 7, 11 and 12, had moderate to high proportions of members from two of the original populations. Clusters 11 and 12 were essentially a mixture of animals from populations 5 and 14 and populations 7 and 19, respectively. Cluster 5 seems to be the most heterogeneous group, containing moderate proportions of animals from populations 1, 9, 2 and 6.

**Table 5:** Percentages of animals at each pre-defined population (20) of Guadarrama goats and F_ST _mean values in each of the 16 clusters inferred.

**assigned to collected from**	**1**	**2**	**3**	**4**	**5**	**6**	**7**	**8**	**9**	**10**	**11**	**12**	**13**	**14**	**15**	**16**	**sample size**
**17**	**54**	2	4	7	4	2	3	3	1	2	3	3	5	3	2	3	187
**1**	3	**38**	2	5	25	2	2	2	2	4	4	2	2	3	2	2	720
**9**	13	**11**	9	6	10	3	3	4	2	3	4	3	5	10	6	6	272
**8**	4	2	**46**	3	3	2	2	2	6	4	7	4	5	4	3	5	219
**11**	6	5	4	**51**	5	2	2	2	2	3	6	3	2	3	2	3	305
**2**	7	7	5	8	**22**	3	2	8	3	3	3	5	4	5	11	4	147
**15**	1	1	1	1	1	**74**	5	2	1	1	2	2	1	2	1	2	570
**4**	2	2	2	1	2	20	**48**	6	2	2	2	2	2	2	2	4	517
**12**	2	2	2	1	2	6	**66**	3	2	2	2	3	2	2	1	3	362
**10**	3	1	1	1	1	3	3	**71**	1	1	2	1	2	3	2	2	141
**13**	8	3	6	5	3	2	2	3	**42**	4	7	3	4	3	2	3	687
**6**	4	3	5	4	9	2	3	3	3	**38**	7	4	5	4	4	3	491
**5**	5	3	13	6	4	3	3	2	5	4	**35**	4	6	3	2	3	326
**14**	6	4	6	12	6	6	1	11	2	7	**20**	3	3	7	2	4	183
**7**	5	3	3	3	3	2	2	5	3	4	3	**40**	4	14	4	3	182
**19**	4	2	5	3	4	2	3	3	12	3	4	**41**	4	4	3	4	264
**3**	7	2	6	4	4	2	2	3	3	4	3	4	**46**	6	2	4	411
**18**	2	1	2	2	3	1	1	5	1	2	2	2	3	**68**	3	2	154
**20**	2	2	1	1	2	1	1	2	1	1	1	2	1	2	**79**	1	218
**16**	2	2	2	2	2	6	3	2	2	2	3	3	4	3	1	**61**	279
**Mean F_ST_**	0.093	0.135	0.099	0.105	0.106	0.205	0.175	0.114	0.108	0.128	0.095	0.106	0.102	0.130	0.161	0.137	6635

Values of the replicate with the highest value of the log probability of data (L(K))

Table 6 shows locus by locus AMOVA analysis which was performed considering groups (16 clusters) and populations (20) as sources of variation. Percentages of variation of the number of alleles (F_ST_) and of the allele size (Rst) among groups, among populations within groups and within populations were estimated. In both cases, the highest percentage of variation (92-93%) corresponded to the within population component. Components among groups and among populations within groups showed low and similar magnitudes (3-4%).

**Table 6:** Locus by locus AMOVA analysis considering groups (16) and populations (20) as sources of variation.

**FST**			
**Source of variation**	**Sum of squares**	**Variance components**	**Percentage of variation**
Among groups	2431.708	0.14910	3.83565
Among populations within groups	282.013	0.14419	3.70938
Within populations	39332.000	3.59399	92.45496
Total			

**RST**			
**Source of variation**	**Sum of squares**	**Variance components**	**Percentage of variation**

Among groups	416659.723	24.69192	3.97462
Among populations within groups	40985.002	17.43235	2.80606
Within populations	6893964.834	579.11560	93.21932
Total	7351609.559	621.23987	

FST = number of different alleles; RST = squared size difference.

## Discussion

In order to maintain genetic diversity, breeding strategies that increase effective population size minimizing genetic drift effect should be implemented. Microsatellite markers in combination with recent statistical methodologies represent a useful tool for the conservation and management of endangered breeds.

A breeding program focused on improving Guadarrama goats milk yield has been carried out in Spain since 1998. In the present work, the actual situation concerning genetic diversity and population structure of this breed has been evaluated using the molecular information derived from 10 microsatellites loci and the use of clustering methods.

The total number of alleles per locus in the present study ranged from 9 to 36. This fact suggested that all markers used were appropriated to analyze genetic diversity in this breed. A more appropriate measure of genetic variation within a population was gene diversity (average expected heterozygosity). Gene diversity estimated in this breed was 0.70, which was in the range (0.3 to 0.8) to be useful for measuring genetic variation [30]. This value was similar to those previously reported (0.69) in other goat breeds [31] and in 31 animals from Guadarrama breed using 30 microsatellites [32]. The mean number of alleles found here (17) was higher than those, 7.7, estimated by Cañón et al. [32]. This could be due to the higher sample size used in our study. In assessing diversity estimates from different studies, it should be mentioned that the values are not directly comparable, as different microsatellite have been used. There were two common microsatellites with Cañon et al. [32]. Hence the comparison has only suggestive indication.

Although only a seven percent of the total genetic variability could be attributed to differences among subpopulations, evidences of a moderate genetic subdivision (mean F_ST _= 0.074) in the Guadarrama goat population were detected. Similar F_ST _value was found in a large analysis [32] using samples of 45 goat breeds from Europe and Middle Eastern countries. Thus, genetic variability within breeds seems to be as important as genetic variability among them. In the Guadarrama goat breed significant genetic differentiation (p < 0.05) was found in 92 out of the 190 population pairs after sequential Bonferroni correction.

The high genetic diversity observed in a breed could be explained by overlapping generations, mixing of populations from different geographical locations, natural selection favouring heterozygosity or subdivision accompanied by genetic drift [33]. Isolation, founder effects, genetic drift and different selection pressures realized by farmers in each population may have played major role in differentiation of Guadarrama goats.

STRUCTURE and BAPS clustering software have the ability of inferring the correct number of subpopulations and assigning individuals appropriately even when genetic differentiation among groups is low (0.02 to 0.05) [34] and using a relative small number of loci (7 microsatellites) [25]. In this case, results derived from both programs provide a strong support of a 16 cluster subdivision. This subdivision seems to be reasonable, since few farmers exchange animals and therefore these populations show more genetic homogeneity. The high average percentage of assignment (77%) of individuals to the population they were collected from, pointed out the existence of clear genetic differences between populations. In addition, AMOVA indicated that 7.5% of the total genetic variation is between populations of this breed while the remaining 92.5% corresponded to differences among individuals.

Genetic differences were not correlated with geographic distances among populations (Mantel test) therefore management factors such as the limited exchange of animals between farmers mostly due to sanitary, social and cultural reasons could constitute the major causes affecting Guadarrama goat population subdivision. In this breed tuberculosis and paratuberculosis are the main causes of mortality and culling. These kinds of diseases have high prevalence in the affected herds. Thus, subdivision would be an advantage preserving the breed from the dissemination of such diseases. Reproductive isolation, consequence of the local use and management of the breed, reduces the effective population size and contributes to the genetic subdivision. Considering Wright' *F-statistics *results, subdivision processes more than inbreeding (average F_IS _across loci was 0.022 ± 0.017), could be the cause of the observed genetic differences between populations. Furthermore, populations analyzed were not in HW equilibrium, as it is revealed by the smaller observed than expected heterozygosity. The heterozygote deficiency is probably reflecting a subdivided population structure (Wahlund effect) rather than selection against heterozygotes.

## Conclusion

In this work we have demonstrated that Guadarrama goat genetic diversity is still conserved. Management factors such as the limited exchange of animals between farmers (estimated gene flow Nm = 3.08) could be the major causes affecting Guadarrama goat population subdivision. Since diseases are the first cause affecting Guadarrama goat census, population subdivision would be an advantage for the conservation of the breed. In such cases, additional constraints, such as the minimum levels of contribution of each population should be included in the conservation strategy [35]. To maintain private alleles present at low frequencies in such small populations avoiding an increase of the inbreeding rate, it would be necessary to develop some strategies to spread such alleles across populations. Since molecular markers allow inferring genealogical relationships, it would be possible to take measures on the mating scheme to minimize co-ancestry or kinship in the subdivided population. The systematic use of molecular markers can facilitate the comprehensive management of endangered populations and should be combined with breeding schemes to improve economic traits avoiding the deterioration of the breeds.

## Authors' contributions

MSN has developed the design of the work, the statistical analyses and wrote the manuscript. JHC has contributed in markers selection and genotyping design, and in manuscript revision. MM has made the animal genotyping and has contributed in the paper discussion. AMC has collaborated in manuscript writing, and results interpretation. FJC has contributed in animal genotyping. CC has collaborated in some technical aspects. JJJ has contributed in the statistical analyses. PDT is the breeder association secretary and has developed the sample collection.

All authors have been read and approved the final manuscript.
